# Peanut, soy, and emerging legume allergy in Canada

**DOI:** 10.1016/j.jacig.2022.05.008

**Published:** 2022-07-20

**Authors:** Josie C.E. Cosyns, Tara Lynn M. Frykas, Hailey V. Hildebrand, Harold Kim, Jennifer D. Gerdts, Elissa M. Abrams, Jennifer L.P. Protudjer

**Affiliations:** a Department of Medicine, University of Manitoba, Winnipeg, Manitoba, Canada; b Department of Food and Human Nutritional Sciences, University of Manitoba, Winnipeg, Manitoba, Canada; i Department of Pediatrics and Child Health, University of Manitoba, Winnipeg, Manitoba, Canada; c Children’s Hospital Research Institute of Manitoba, Winnipeg, Manitoba, Canada; d Canadian Society of Allergy and Clinical Immunology, Orleans, Ontario, Canada; e Division of Clinical Immunology and Allergy, Western University, London, Ontario, Canada; f Division of Clinical Immunology and Allergy, McMaster University, London, Ontario, Canada; g Food Allergy Canada, Toronto, Ontario, Canada; h Division of Allergy and Immunology, Department of Pediatrics, University of British Columbia, Vancouver, British Columbia, Canada; j George and Fay Yee Centre for Healthcare Innovation, Winnipeg, Manitoba, Canada; k Centre for Allergy Research, Karolinska Institutet, Stockholm, Sweden

## Abstract

**Background:**

Individuals with 1 legume allergy may be cosensitized to other legumes and thus may potentially have other legume allergies as well. Although the use of emerging legumes (eg, pea, lentils, chickpeas) in commercial food production is increasingly common, the literature has largely focused on peanut and soy, both of which are priority allergens in Canada.

**Objective:**

We aimed to describe the distribution of priority and emerging legume allergies in Canada, with consideration for patient age.

**Methods:**

Cross-sectional survey data collected between 2019 and 2021 from families who follow food allergy–related social media platforms were queried for demographics, as well as for food allergy (including by type and number of foods and by age [0-5 vs ≥6 years]). Data were described and then analyzed by using logistic regression and adjusted for sex, age at diagnosis, and number of food allergies.

**Results:**

Of the 115 participating children, the majority (64.6%) were boys. Nearly all of the children (109 of 115 [94.8%]) had peanut allergy, whereas soy and emerging legume allergies were reported by 15.7% and 13.0% of the children, respectively. Of these 115 children, 85 had mono-peanut allergy, 6 had mono-soy allergy, none had emerging legume allergy in the absence of peanut or soy, 12 had peanut and emerging legume allergy, 9 had peanut and soy allergy, and 3 had peanut, soy, and emerging legume allergy. Compared with children aged 0 to 5 years, children aged 6 years or older were significantly less likely to have peanut plus soy or emerging legume allergy (odds ratio = 0.22 [95% CI = 0.05-0.94]; *P* = .04).

**Conclusion:**

Of the children with peanut allergy, a considerable number also had peanut allergy and soy allergy and/or another legume allergy. Younger children have higher odds of multiple legume allergy.

## Introduction

Individuals with peanut allergy may be cosensitized to 1 or more other legumes, potentially resulting in coallergy.^1^ Studies propose a moderate, between 29% and 35%, prevalence of symptoms in response to other legumes after ingestion in individuals with peanut allergy.^2^ Yet, much existing work has been restricted to peanut and soy—not to other legumes. In a scoping review, only 7 studies reporting on legume allergies other than soy allergy and peanut allergy came from North America.^3^ There is little evidence about the distribution of peanut and soy allergies (priority allergens) and emerging legume allergens in Canada. Thus, we aimed to describe the distribution of priority and emerging legume allergies in Canada with consideration for patient age.

Parents of children with at least 1 of 6 legume allergies (ie, peanut, soy, lentil, pea, chickpea, or lupine allergy) completed an online, anonymous survey. The survey was developed on the basis of 2 previously validated questionnaires: the Food Allergy Quality of Life Questionnaire^4^, ^5^, ^6^ and the Food Allergy Socio-Economic Questionnaire,^7^^,^^8^ as well as on the basis of the study team’s *a priori* knowledge of the field.

Data were collected as part of 2 different studies, MultidemeNsional bUrden of Allergies iN Canadian childrEn and adultS Households, which studied households with at least 1 child with multiple food allergies (conducted from 2019 to March 2020 and circulated via Food Allergy Canada, which is our national patient organization) and Food Allergy, Racial-ethnic Identity and food insecurity (conducted during the COVID-19 pandemic and circulated via social media, including “tagging” Food Allergy Canada). Both studies were approved by the University of Manitoba Health Research Ethics Board.

Priority legume allergies were defined as peanut and/or soy allergy.^9^ Emerging legume allergies included any legume allergy other than peanut or soy allergy, per parent-completed open-text responses. Specific emerging legume allergens included chickpea, pea, lentils, and nonspecified legumes other than peanut or soy. Age groups were collapsed into binary categories of 0 to 5 years and 6 years or older. Data were described as numeric values and percentages and presented as proportional Venn diagrams and then analyzed using logistic regression (reported as odds ratios and 95% CIs) and adjusted for sex, age at diagnosis, and total number of food allergies, with statistical significance set at a *P* value less than .05. Analysis was performed using Stata software, version 16 (StataCorp, College Station, Tex).

## Results and discussion

**Fig 1:**
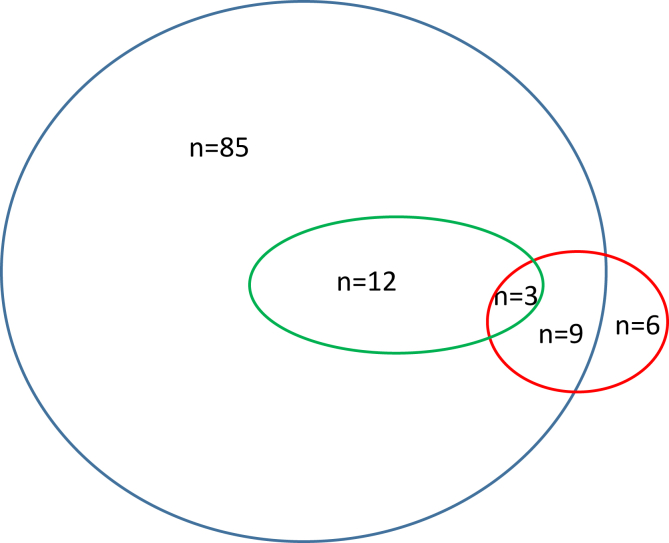
Proportional Venn diagram of the distribution of peanut allergy (*blue*), soy allergy (*red*), and emerging legume allergies (*green*).

Our study population included 115 children from all Canadian provinces; they were disproportionately boys (64.6%), and one-third of them were aged 6 years or younger (see Table E1 in the Online Repository at www.jaci-global.org). Of the 115 children, 109 (94.8%) had peanut allergy, with lower prevalences of soy (18 of 115 [15.7%]) and emerging legume (15 of 115 [13.0%]) allergies. Most of the children (106 of 111 [95.5%]) had an epinephrine autoinjector and had been diagnosed by an allergist (96 of 99 [98.0%]). Of these 115 children, 85 had mono-peanut allergy, 6 had mono-soy allergy, none had emerging legume allergy in the absence of peanut or soy, 12 had peanut and emerging legume allergy, 9 had peanut and soy allergy, and 3 had peanut, soy, and emerging legume allergy (Fig 1). Of the 15 children with emerging legume allergies, 9 (60%) had been diagnosed by oral food challenge. Compared with children aged 0 to 5 years, children aged 6 years or older were significantly less likely to have peanut plus soy or an emerging legume allergy (odds ratio = 0.22 [95% CI = 0.05-0.94]; *P* = .04) (Table I), whereas these differences were attenuated when priority versus priority plus emerging legume allergies were considered. Among the children with emerging legume allergies, there was no clear pattern between type of emerging legume allergy and comorbid priority legume (ie, peanut and soy) allergy (see Table E2 in the Online Repository at www.jaci-global.org).

**Table I:** Logistic regression analyses of priority versus emerging legume allergies

Allergy type			Unadjusted		Adjusted∗	
	No.	%	OR	95% CI	OR	95% CI
Priority vs priority + emerging legumes						
0-5 y	67	58.3	Ref		Ref	
≥6 y	48	41.7	1.26	0.42; 3.74	0.71	0.17; 3.03
Peanut only vs peanut + other legume(s)						
0-5 y	62	56.9	Ref		Ref	
≥6 y	47	43.1	0.59	0.23; 1.52	**0.22**†	**0.05; 0.94**

Priority legume allergy is defined as peanut or soy allergy. Emerging allergy is defined as any legume other than peanut or soy. Boldface indicates statistical significance.

*OR*, Odds ratio; *Ref*, reference.

∗ Adjusted for sex, age at diagnosis, and total number of food allergies.

† *P* < .05.

This is the first ever Canadian description of priority and emerging legume allergies with respect to age. The overwhelming majority of children with an emerging legume allergy had peanut allergy, and that peanut allergy was a predictor for comorbid emerging legume allergy. Males and children aged 0 to 5 years were disproportionately affected. Likewise, younger children were more likely than older children to have multiple allergies.

Peanut is one of the most common allergens affecting children in the Western world, accounting for approximately 2% of the population having the allergy.^10^^,^^11^ Nearly all of the participants in our study had peanut allergy, with age and sex distributions consistent with those in previous studies.^10^^,^^12^, ^13^, ^14^ Our study results show that prevalences of soy and emerging legume allergies were lower than the prevalence of peanut allergy. However, at 15.7% and 13%, respectively, the rates were higher than expected according to other reports.^10^ In addition, we demonstrated that children between the ages of 0 and 5 years were more likely to have peanut plus at least 1 other legume allergy, suggesting that peanut allergy may predispose young children to development of subsequent legume coallergy. The decrease in coallergy seen in older children may be attributed to differences in the rate of allergy resolution. Soy allergy, for example, has a resolution rate of 45% by the age of 6 years and continues to resolve into adolescence, whereas peanut allergy has a resolution rate of approximately 20% by the age of 4 years and is more commonly a lifelong, chronic condition.^13^

Regional variations in legume consumption may play a role in the prevalence, cosensitization, and cross-reactivity of various legume allergies. Peanut allergy has been reported to be the most common legume allergy in the United Kingdom, France, Switzerland, and North America.^3^ In contrast, soybean predominates in Japan, lentil and chickpea predominate in Spain, and lupine predominates in various regions of Europe.^1^

Notably, the European Union mandates that some emerging legume allergens, such as lupine, must be declared on prepackaged foods.^14^ At present, no such labeling requirements exist in Canada.^3^ However, in Canada, 11 allergens, including peanut and soy, must be included in the ingredients list of prepackaged food labels.^3^ Allergy labeling is recognized as an important risk management tool for children with food allergy and their families, who rely on strict allergen avoidance. Emerging legume allergens have been reported to elicit severe allergic reactions and studies suggest a moderate rate of cross-reactivity between priority and emerging legumes, as was also demonstrated in our study.^1^ Findings in our study highlight the importance of identifying emerging legume allergies in Canada to implement proper legume allergy education and more inclusive food allergy labeling requirements to prevent accidental exposure and improve the quality of life of individuals with legume allergy and their caregivers.

We acknowledge that in our study legume allergy was based on parent report. However, nearly all of the children had been diagnosed by an allergist and carried an epinephrine autoinjector—and thus were likely to truly have an allergy.

In conclusion, of the children with peanut allergy, a considerable number also had peanut allergy and soy allergy and/or another legume allergy. One-third of all individuals with legume allergy were younger than 6 years and were predominantly male.

**Clinical implications**

Of the children with peanut allergy, a considerable number also had soy and/or another legume allergy. One-third of all individuals with legume allergy were younger than 6 years and most were male.

## Supplementary data

Table E1

Table E2
