# Supplementary material for: Peanut, soy, and emerging legume allergy in Canada
Source: J Allergy Clin Immunol Glob. 2022 Jul 20;1(4):319–21. doi: 10.1016/j.jacig.2022.05.008 (PMC10509839; doi:10.1016/j.jacig.2022.05.008)
Supplement: Table E1 [file mmc1.docx]

| **Table E1**. Participant Characteristics (N=115) | | |
| --- | --- | --- |
|  |  |  |
|  | **n** | **%** |
| **Demographics** |  |  |
| Age group (years; N=104) |  |  |
| 0-2 | 17 | 16.3 |
| 3-6 | 18 | 17.3 |
| 7-11 | 32 | 30.8 |
| 12-14 | 25 | 24.0 |
| 15-17 | 12 | 11.5 |
| Sex (N=113) |  |  |
| Boys | 73 | 64.6 |
| Girls | 40 | 35.4 |
| Region |  |  |
| Eastern Canada* | 59 | 51.3 |
| Western Canada† | 51 | 44.4 |
| Maritimes‡ | 5 | 4.4 |
| **Allergy characteristics** |  |  |
| Food allergies§ |  |  |
| Peanut | 109 | 94.8 |
| Soy | 18 | 15.7 |
| Non-priority legumes | 15 | 13.0 |
| Tree nuts | 86 | 74.8 |
| Sesame | 40 | 34.8 |
| Milk | 50 | 43.5 |
| Egg | 63 | 54.8 |
| Fish | 24 | 20.9 |
| Crustaceans | 23 | 20.0 |
| Wheat | 8 | 7.0 |
| Mustard | 8 | 7.0 |
| Other | 22 | 19.1 |
| Age (years) at diagnosis |  |  |
| 0-2 | 13 | 11.3 |
| 3+ | 12 | 10.4 |
| Allergist-diagnosed (N=98) | 96 | 98.0 |
| SPT, Blood test | 110 | 95.7 |
| OFC (N=114) | 63 | 55.3 |
| EAI (N=111) | 106 | 95.5 |
|  |  |  |
| *Provinces of Ontario and Quebec  †Provinces of British Columbia, Alberta, Saskatchewan, and Manitoba |  |  |
| ‡NS, PE, NB, NL |  |  |
| §Not mutually exclusive |  |  |
|  |  |  |

Abbreviations: *EAI* Epinephrine autoinjector; *OFC* Oral food challenge*; SPT* Skin prick test
