# Supplementary material for: Peanut, soy, and emerging legume allergy in Canada
Source: J Allergy Clin Immunol Glob. 2022 Jul 20;1(4):319–21. doi: 10.1016/j.jacig.2022.05.008 (PMC10509839; doi:10.1016/j.jacig.2022.05.008)
Supplement: Table E2 [file mmc2.docx]

| **Table E2.** Distribution of non-priority legume allergies, by type of legume and priority legume allergy (N=15 participants) | | | | | | | |
| --- | --- | --- | --- | --- | --- | --- | --- |
|  |  |  |  |  |  |  |  |
|  | **Non-priority legumes** | | | | | **Priority legumes** | |
| Participant | Chickpea | Pea | Lentils | Unspecified | Total | Peanut | Soy |
| 1 | 1 | 1 | 1 |  | 3 | 1 |  |
| 2 | 1 | 1 | 1 |  | 3 | 1 |  |
| 3 |  | 1 | 1 | 1 | 3 | 1 | 1 |
| 4 | 1 | 1 |  |  | 2 | 1 |  |
| 5 | 1 | 1 |  |  | 2 | 1 |  |
| 6 | 1 |  |  |  | 1 | 1 |  |
| 7 | 1 |  |  |  | 1 | 1 |  |
| 8 |  | 1 |  |  | 1 | 1 |  |
| 9 |  | 1 |  |  | 1 | 1 |  |
| 10 |  | 1 |  |  | 1 | 1 |  |
| 11 |  | 1 |  |  | 1 | 1 |  |
| 12 |  | 1 |  |  | 1 | 1 |  |
| 13 |  | 1 |  |  | 1 | 1 | 1 |
| 14 |  |  |  | 1 | 1 | 1 | 1 |
| 15 |  |  |  | 1 | 1 | 1 |  |
